# Familial hypertrophic cardiomyopathy: functional variance among individual cardiomyocytes as a trigger of FHC-phenotype development

**DOI:** 10.3389/fphys.2014.00392

**Published:** 2014-10-10

**Authors:** Bernhard Brenner, Benjamin Seebohm, Snigdha Tripathi, Judith Montag, Theresia Kraft

**Affiliations:** Institute of Molecular and Cell Physiology, Hannover Medical SchoolHannover, Germany

**Keywords:** *MYH7*-mutations, allelic imbalance, myocyte disarray, interstitial fibrosis, converter domain of myosin head, myosin head stiffness, calcium sensitivity of muscle fibers, calcium sensitivity of cardiomyocytes

## Abstract

Familial hypertrophic cardiomyopathy (FHC) is the most frequent inherited cardiac disease. It has been related to numerous mutations in many sarcomeric and even some non-sarcomeric proteins. So far, however, no common mechanism has been identified by which the many different mutations in different sarcomeric and non-sarcomeric proteins trigger development of the FHC phenotype. Here we show for different *MYH7* mutations variance in force pCa-relations from normal to highly abnormal as a feature common to all mutations we studied, while direct functional effects of the different FHC-mutations, e.g., on force generation, ATPase or calcium sensitivity of the contractile system, can be quite different. The functional variation among individual *M. soleus* fibers of FHC-patients is accompanied by large variation in mutant vs. wildtype β-MyHC-mRNA. Preliminary results show a similar variation in mutant vs. wildtype β-MyHC-mRNA among individual cardiomyocytes. We discuss our previously proposed concept as to how different mutations in the β-MyHC and possibly other sarcomeric and non-sarcomeric proteins may initiate an FHC-phenotype by functional variation among individual cardiomyocytes that results in structural distortions within the myocardium, leading to cellular and myofibrillar disarray. In addition, distortions can activate stretch-sensitive signaling in cardiomyocytes and non-myocyte cells which is known to induce cardiac remodeling with interstitial fibrosis and hypertrophy. Such a mechanism will have major implications for therapeutic strategies to prevent FHC-development, e.g., by reducing functional imbalances among individual cardiomyocytes or by inhibition of their triggering of signaling paths initiating remodeling. Targeting increased or decreased contractile function would require selective targeting of mutant or wildtype protein to reduce functional imbalances.

## Introduction

Familial hypertrophic cardiomyopathy (FHC) is the most frequent inherited cardiac disease and the most common cause of sudden cardiac death in otherwise healthy young individuals and athletes (Maron et al., 2003). FHC is characterized by asymmetric hypertrophy of the left ventricle, pronounced myocyte and myofibrillar disarray, and interstitial fibrosis. These structural features together with arrhythmias, unexplained syncopes and sudden cardiac death are hallmarks of the FHC. The clinical phenotype of FHC is heterogeneous ranging from almost asymptomatic to highly malignant with sudden cardiac death or development of end-stage heart failure (Spirito et al., 1987; Maron et al., 2003). More than 300 FHC-related mutations were identified within the in the β-myosin heavy chain (β-MyHC; Moore et al., 2012) revealing allelic genetic heterogeneity. FHC-related mutations, however, were also found in a large number of other sarcomeric proteins (non-allelic genetic heterogeneity in FHC), few even in non-sarcomeric proteins. Mutations in the β-MyHC, cardiac myosin-binding protein C (cMyBPC), cardiac troponin-T (cTnT), and cardiac troponin-I (cTnI) account for nearly 90% of all FHC-cases. About 40% of all genotyped FHC-patients carry missense mutations in *MYH7*, about 30–40% in the cMyBPC gene (Richard et al., 2003; Fokstuen et al., 2008; Ho et al., 2010). So far, however, it is still unclear how altogether several hundred different mutations in a large number of different sarcomeric and some non-sarcomeric proteins result in the characteristic features of FHC.

Since FHC is a monogenic disease, the phenotype is thought to result from the triggering of phenotype development by the respective mutation (Ashrafian et al., 2011). This raised the question about the trigger common to all the different mutations in the different proteins. *In vitro* motility and ATPase-assays on isolated sarcomeric proteins together with the analysis of mouse models led to the hypothesis that enhanced calcium-sensitivity, increased maximal force generation, and higher ATPase activity are the common features of FHC-related mutations (Robinson et al., 2002, 2007; Debold et al., 2007), resulting in impaired energy metabolism (Spindler et al., 1998; Blair et al., 2001) and altered calcium-handling in cardiomyocytes (Baudenbacher et al., 2008; Guinto et al., 2009). Several data reported about functional effects of FHC-mutations are in conflict with this hypothesis. For example, force generation of cardiomyocytes from tissue samples of affected patients was reduced compared to control for β-MyHC mutations, mutations in the cMyBPC, and for FHC-patients with unidentified mutations (Hoskins et al., 2010; van Dijk et al., 2012; Kraft et al., 2013). For several β-MyHC mutations calcium sensitivity was found reduced, or unchanged but with residual active forces under relaxing conditions (Kirschner et al., 2005; Kraft et al., 2013). For only two out of four β-MyHC mutations ATPase was enhanced but unchanged for the others (Seebohm et al., 2009; Witjas-Paalberends et al., 2014) while two out of three β-MyHC mutations showed higher force generation than controls while force generation was unchanged for the third when force generation was studied in *M. soleus* fibers of affected patients (Seebohm et al., 2009). Thus, the effects of quite many FHC-mutations do not fall into the previously proposed common mechanism for FHC-development of increased contractile functions. This could, in part, be due to secondary effects like myofibrillar disarray affecting some of these parameters, e.g., maximum force generation (Kraft et al., 2013). Thus, altogether no common trigger for FHC-development has been identified so far. Knowing the trigger and subsequent steps in the pathogenesis of FHC holds the potential to identify novel targets for novel therapeutic strategies, e.g., in the prevention cardiac remodeling in FHC-patients harboring different FHC-related mutations.

Here we summarize our work on the functional characterization of FHC-related mutations in the β-MyHC both in skeletal and myocardial tissue samples of affected patients. Our goal was to identify features that might be common to many if not all FHC-related mutations and thus may be a trigger for development of the typical FHC-phenotype by different mutations in different sarcomeric and even non-sarcomeric proteins. We will finally discuss a possibly common feature and how it might initiate myocyte disarray, interstitial fibrosis and hypertrophy, the hallmarks of FHC-related cardiac remodeling.

## Effects of FHC-related mutations in the β-MyHC on force generation and fiber stiffness

### Measurements on isolated fibers of *M. soleus* tissue samples of FHC-patients

**Figure 1:**
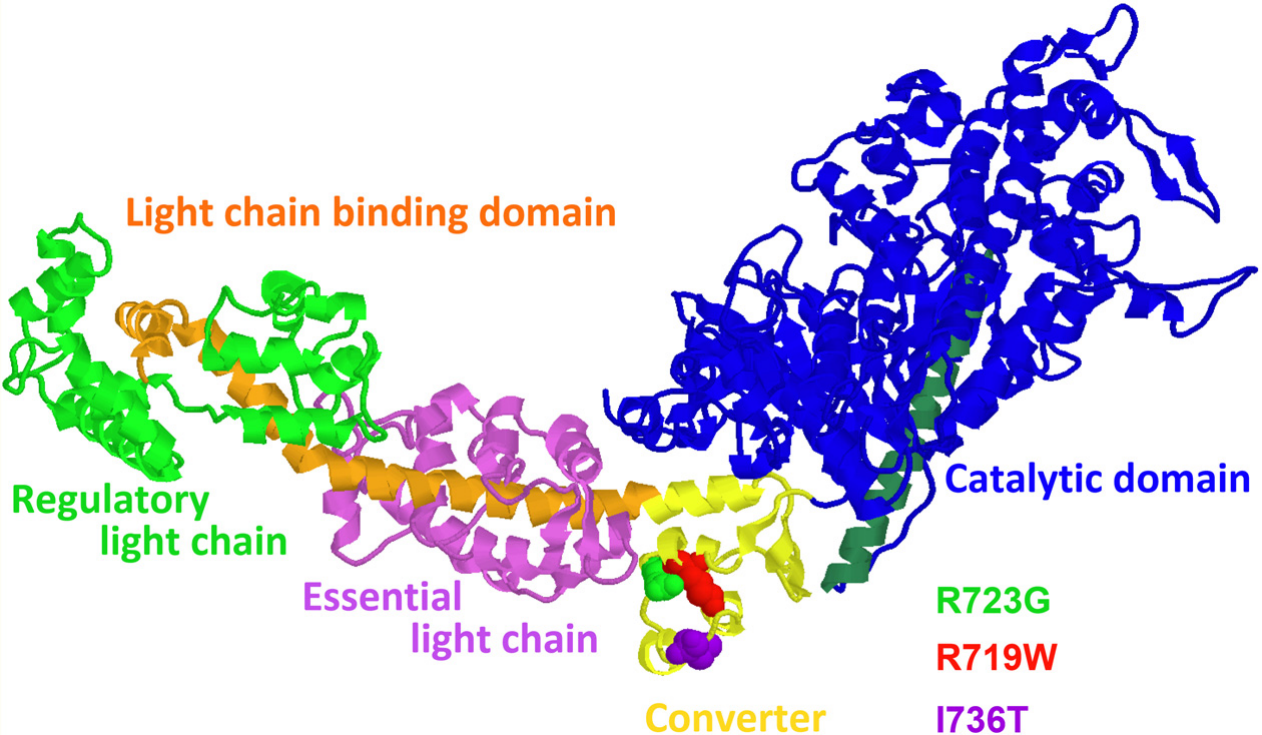
**Structural model of the myosin head domain (Rayment et al., 1993) illustrating the locations of the converter mutations R719W, R723G, and I736T**. Note location of mutations R719W and R723G more in the core of the converter in a helix close to the long α-helix of the light chain binding domain that is anchored in the converter. Mutation I736T is near the surface of the converter (figure prepared with RasMol).

We had focussed our earlier work on the functional effects of missense mutations in the converter domain of the β-MyHC, mutations R719W, R723G, and I736T (cf. Figure 1, Rayment et al., 1993). Our goal was to identify direct functional effects of these mutations on muscle function that may be common to all three mutations and common to other FHC-related mutations, including mutations in other proteins.

Tissue samples of *M. soleus* were obtained by open biopsy. The samples were immediately separated in small bundles and fiber membranes were dissolved by incubation in skinning solution, an ATP-containing solution mimicking the intracellular ionic milieu to which 0.5% Triton X100 was added. Fiber bundles were then equilibrated with solutions containing increasing concentrations of sucrose (maximum 2 M) as a cryoprotectant. Fiber bundles were then rapidly frozen in liquid propane and stored in liquid nitrogen until use. For details see Kraft et al. (1995). For experiments fiber bundles were thawed, individual fibers were isolated and mounted between a strain gage force transducer and a motor to control muscle length or load. As a direct measure of length and length changes of the muscle fibers we measured sarcomere length by laser light diffraction. For further details see Seebohm et al. (2009). The mounted individual fibers were bathed in different experimental solutions that mimicked the ionic composition of the intracellular medium. Different free Ca^++^-ion concentrations were obtained by adding the calcium chelator EGTA and CaEGTA in appropriate proportions. Free Ca^++^-ion concentrations were calculated according to Föhr et al. (1993). For measurements under rigor conditions, fibers were incubated in solutions without MgATP that contained 3 mM EDTA to quickly remove free Mg^++^-ions.

**Figure 2:**
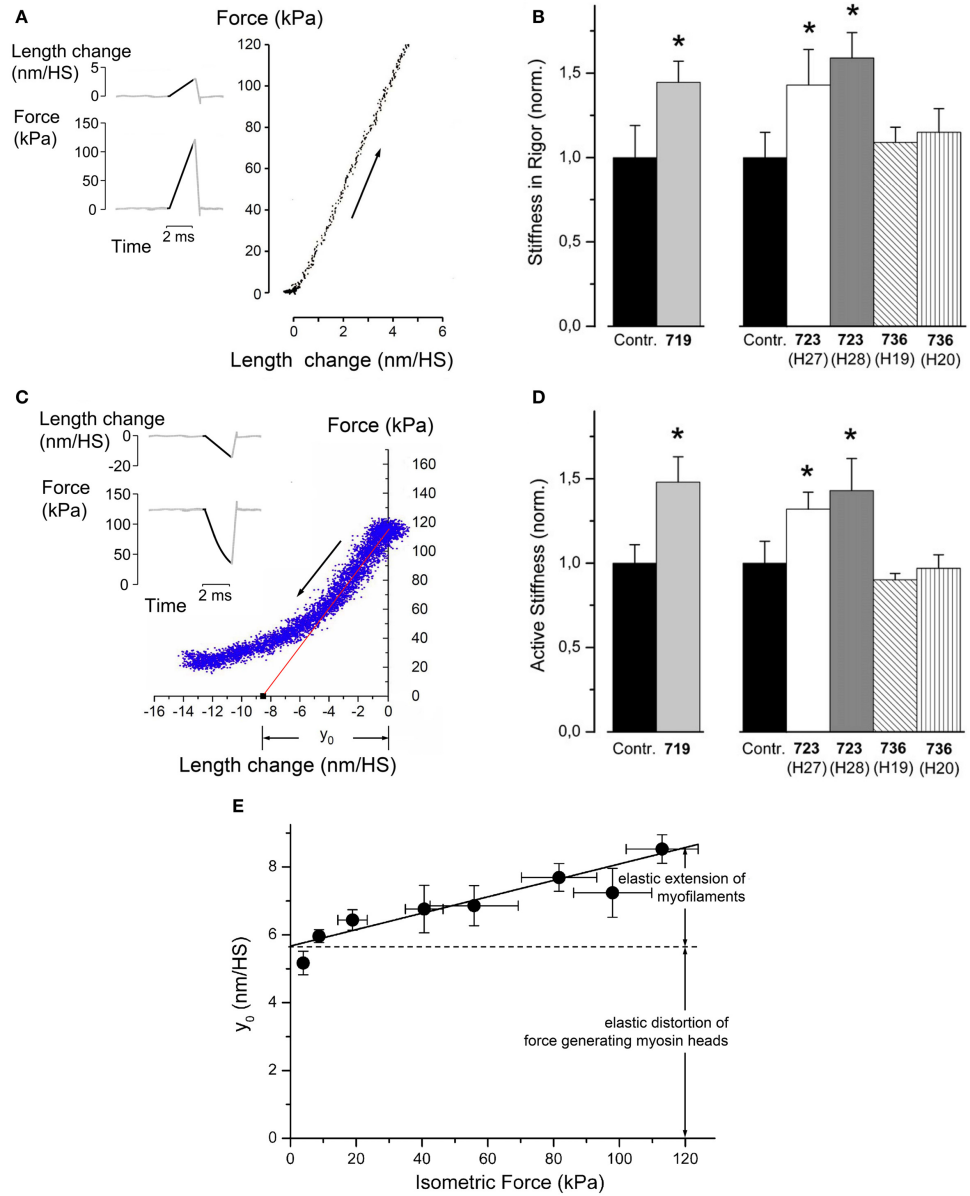
**Effects of mutations R719W, R723G, and I736T on stiffness of soleus muscle fibers in rigor (absence of nucleotide) and during active contraction**. **(A)** Principle of stiffness measurement in rigor. In rigor fibers generate no force when put in rigor by rapid removal of free Mg^2+^-ions together with removal of ATP (Brenner et al., 1986). Thus, stiffness was measured by applying a ramp-shaped stretch (see inset for a schematic illustration). Stiffness is the slope when force change is plotted vs. applied change in sarcomere length, measured in nm/half sarcomere (nm/HS). Note that only the dark part of the traces in the inset are included in the force vs. length change plot. Noise of this plot was reduced by signal-averaging responses of several such maneuvers. **(B)** Fiber stiffness in rigor for the mutations and control fibers. Speed of stretch ~3–5 × 10^3^ (nm/half sarcomere)s^−1^; *T* = 5°C; *n* = 5–10 fibers. Mean values ± *SE*, normalized control fibers, respectively. ^*^Difference to controls statistically significant, *p* ≤ 0.05. **(C)** Principle of stiffness measurements during isometric steady state contraction. When fibers have reached constant isometric force a ramp shaped release is imposed on the fiber length. This results in a drop of isometric force (cf. inset for schematic illustration of length change and force response). Over the initial 2–3 nm of length change the plot of force vs. sarcomere length change is linear. Deviation from the linear response later in the release is due to rapid re-equilibration of myosin heads in their different force-generating states. The slope of the initial linear part is determined by linear regression (red line) and taken as active fiber stiffness. The intercept of this line with the abscissa is the *y*_0_-value. This is interpreted as the elastic extension of the actin and myosin filaments plus elastic distortion of the force generating myosin heads (Linari et al., 2007). The plot of force vs. length change is noisier than in **(B)**. This is due to the fact that fewer measurements were signal-averaged than under rigor conditions shown in **(A)**. **(D)** Fiber stiffness determined during isometric steady state contraction [speed of release ~2–4 × 10^3^ (nm/half-sarcomere)s^−1^]; *T* = 10°C to ensure structural integrity of fibers and stability of striation pattern throughout experiments; *n* = 6–14 fibers. Mean values ± *SE*, normalized control fibers, respectively. ^*^Difference to controls statistically significant, *p* ≤ 0.05. **(E)** Plot of y_0_, vs. isometric force at different levels of calcium-activation. The *y*_0_-values were measured from T-plots (cf. **C**) at pCa values from 4.5 to 6.6; *T* = 10°C. Speed of length release 4–5 × 10^3^ (nm/half-sarcomere)s^−1^. Continuous line obtained by linear regression. Y-axis intercept of solid line is 5.67 nm/HS (HS = half sarcomere) equals elastic extension of force generating heads (cf. double headed arrow). Slope of solid line is filament compliance per half-sarcomere, about 0.025 nm/kPa. Elastic extension of myofilaments at an isometric force of 120 kPa is illustrated by double arrow. Panels **(B, D** and **E)** reprinted with modifications from Seebohm et al. (2009) with permission from Elsevier.

We first measured stiffness of single *M. soleus* muscle fibers in rigor (Figures 2A,B) and during active contraction (Figures 2C,D, Seebohm et al., 2009). Stiffness was measured by applying small ramp-shaped length changes, stretches (Figure 2A) or releases (Figure 2C) for stiffness in rigor or during active contraction, respectively. During these length changes force and change in sarcomere length were recorded. Fiber stiffness was measured as the slope when force was plotted vs. change in sarcomere length (cf. Figures 2A,C). For stiffness during active contraction the slope over the initial 2–3 nm of length change was determined (red line in Figure 2C) for further details of stiffness measurements see Brenner (1998). Under rigor conditions and during active contraction we observed an increased fiber stiffness for mutations R723G and R719W, while stiffness of fibers with mutation I736T was unchanged (Figures 2B,C). The increased stiffness in rigor, i.e., when all myosin heads are attached to actin, immediately suggested that mutations R723G and R719W both increase the stiffness of the myosin head domain.

### Quantitative estimate of the change in myosin head stiffness by mutations R719W and R723G

For a quantitative estimate of the increase in stiffness per myosin head we had to determine the elastic distortion of the myosin head vs. elastic extension of myofilaments while active force was generated or while fibers were in rigor. This was possible from measurements of active force and active stiffness at different degrees of Ca^++^-activation (cf. Linari et al., 2007). Figure 2E shows these data and illustrates the separation between elastic head distortion and elastic filament extension at different levels of active force. The slope of the solid line in Figure 2E is the compliance of the myofilaments. The intercept of this line with the ordinate at about 5.5 nm/HS represents the elastic extension of the force-generating cross bridges. This is assumed to be the same for all levels of Ca^++^-activation. The *y*_0_-value is the amplitude of fiber release per half-sarcomere that is necessary to drop active force to zero if no fast redistribution of cross-bridges among their different states had occurred (intercept of red solid line in Figure 2C with abscissa at about 8.6 nm/HS). In this concept the y_0_ equals the elastic extension/distortion of the force generating cross-bridges, about 5.6 nm, plus the elastic extension of the myofilaments that increases with the forces (isometric force) that act on the myofilaments (cf. Linari et al., 2007).

**Figure 3:**
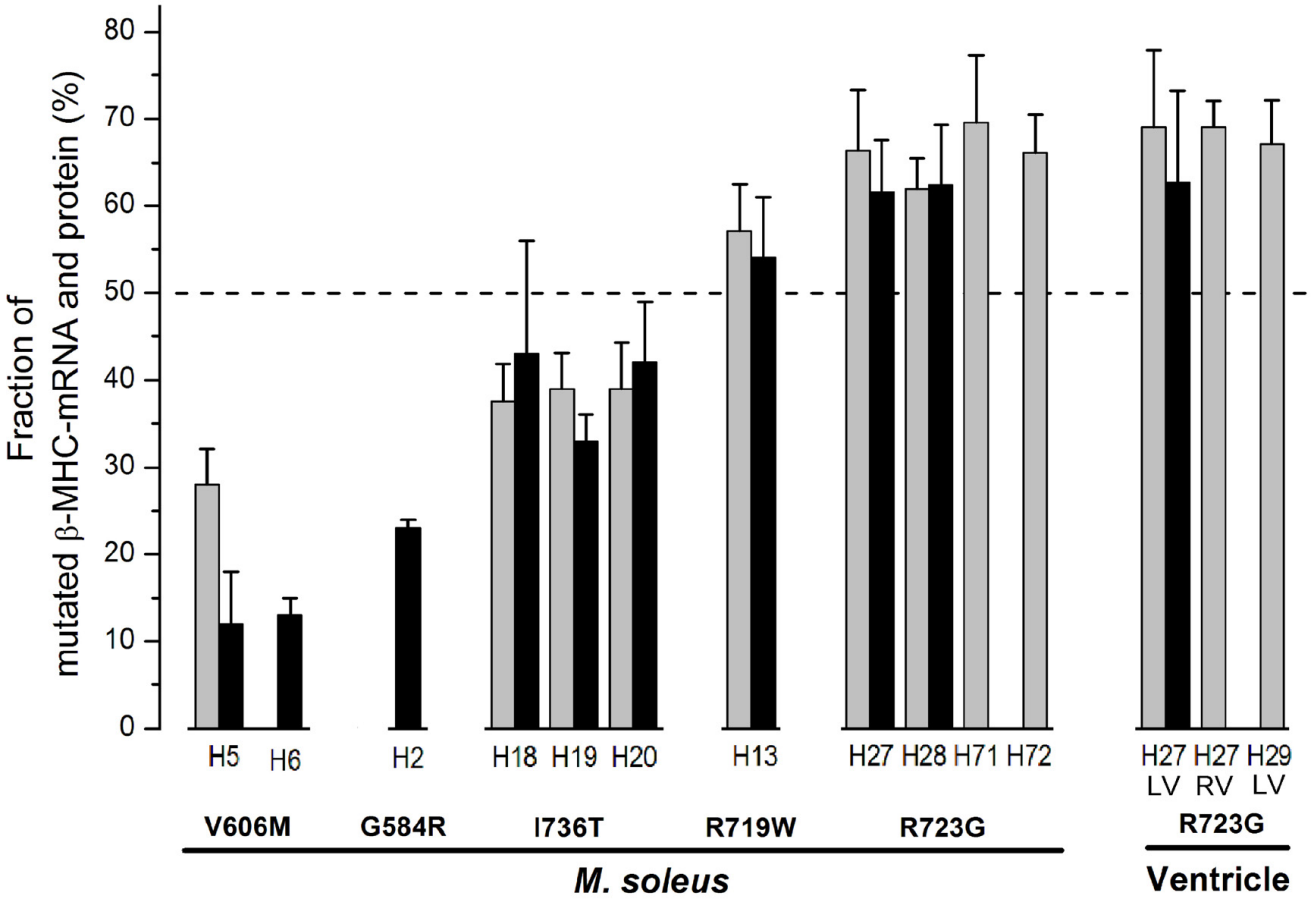
**Fractions of mutated β-cardiac/slow skeletal MyHC-mRNA and of β-cardiac/slow skeletal MyHC protein for different FHC-mutations**. Gray bars, fractions of mutated mRNA; black bars, fractions of mutated protein. Data for *M. soleus* samples of mutations V606M, G584R, I736T, R719W, and R723G. For mutation R723G data from ventricular myocardium are also included (LV, left ventricle; RV, right ventricle). Note that all mutations significantly deviate from equal abundance of wild-type and mutated transcript (allelic imbalance) with very similar deviations also at the protein level. Allelic imbalance for mutated mRNA and protein were very similar in *M. soleus* and myocardium (mutation R723G). Also note the essentially identical expression of mRNA and protein both within families (I736T, R723G) and among unrelated families (V606M, R723G). Reprinted from Tripathi et al. (2011).

For a quantitative estimate of the changes in stiffness and in the contribution to force generation by the individual myosin head, caused by the converter mutations, we also had to know how many myosin molecules actually carry the FHC-mutation. From the autosomal dominant inheritance one may have assumed that 50% of the myosin molecules carry the mutation while the other 50% have the wildtype sequence. Analysis by mass spectrometry, however, revealed that the abundance of mutant protein is not equal to 50% but instead is characteristic for each particular mutation (allelic imbalance; Figure 3, black bars). The same allelic imbalance is found for different members of the same family (cf. I734T, R723G) and for different generations (cf. R723G), but also in unrelated families with the same mutation (cf. V606M, R723G). In addition, the same abundance is found in *M. soleus* fibers and in left ventricular myocardium (cf. R723G). Analysis of expression of the mutant allele at the mRNA-level revealed very similar fractions of mutant mRNA (allelic imbalance) as was found for the mutant protein (Figure 3, gray bars vs. black bars).

The known fraction of mutant myosin in our samples allowed us to estimate the change in head stiffness (change in resistance to elastic distortion) from the observed increase in fiber stiffness, i.e., of the mixture of mutant and wildtype myosin molecules, and the known compliance of the myofilaments (Figure 2C, Seebohm et al., 2009). This estimate yielded an about 2.6-fold increase in head stiffness for mutation R719W and about 2.9-fold increase for mutation R723G while mutation I736T had no such effect (Seebohm et al., 2009).

Based on the fundamental mechanism of force generation, i.e., that active forces result from elastic distortion of actin attached myosin heads caused by structural changes in the myosin head during its working stroke (Huxley, 1957), an increased head stiffness that corresponds to an increased resistance to elastic distortion is expected to result in increased generation of active force. In fact the estimated increase in stiffness of the individual head domain indicated by our stiffness measurements predicted an increase in force generation that was very close to that observed experimentally, about 2-fold for the head domains with mutation R723G or R719W (Seebohm et al., 2009).

The increased head stiffness by converter mutations R719W and R723G implies that the converter domain itself is a major determinant of head stiffness since a point mutation in a particular domain can result in an increase in head stiffness only if the affected domain is a compliant part of the molecule. Stiffening of an already rigid component would not affect overall “stiffness” of a molecule since overall stiffness is limited by the most compliant element(s) of the molecule. But why should increased head stiffness and increased contribution to force generation by an individual myosin head cause disease?

Since mutations R719W and R723G increased myosin head stiffness while mutation I736T did not, we wondered whether the first two mutations were located at particular points in the converter sequence. We therefore compared the amino acid sequence of the β-cardiac myosin heavy chain (which is also the heavy chain of slow skeletal muscle) with the sequences of fast skeletal myosin heavy chains. This revealed that the FHC-mutations R719W and R723G are located at positions where the β-cardiac/slow MHC differs from fast MyHC isoforms in the otherwise highly conserved converter region (cf. Table 1). This raised the question, whether fast and slow skeletal myosins may actually differ in the head stiffness and thus the force generated by a cross-bridge in a force-generating state. We tested this by both stiffness measurements on skinned fibers of human soleus (β-cardiac/slow skeletal MyHC) vs. rabbit psoas (fast skeletal MyHC-2D) and by optical trapping on β-cardiac/slow skeletal myosin subfragment 1 prepared from human and rabbit slow skeletal muscle fibers vs. fast myosin subfragment 1 from the rabbit psoas (Seebohm et al., 2009; Brenner et al., 2012). This revealed that the fast MyHC has ≥3-fold higher head stiffness than the slow/β-cardiac MyHC (cf. Table 1). This was supported by data in the literature (cf. Table 1; Capitanio et al., 2006; Lewalle et al., 2008). So increased head stiffness with increased contribution to force generation appears to be physiological in fast skeletal muscle fibers. This made it even more puzzling as to why converter mutations R719W and R723G cause disease. In addition, increased head stiffness with increased contribution to force generation cannot act as a common trigger for FHC-development, not even for myosin mutations since mutation I736T showed no such effect.

**Table 1:**
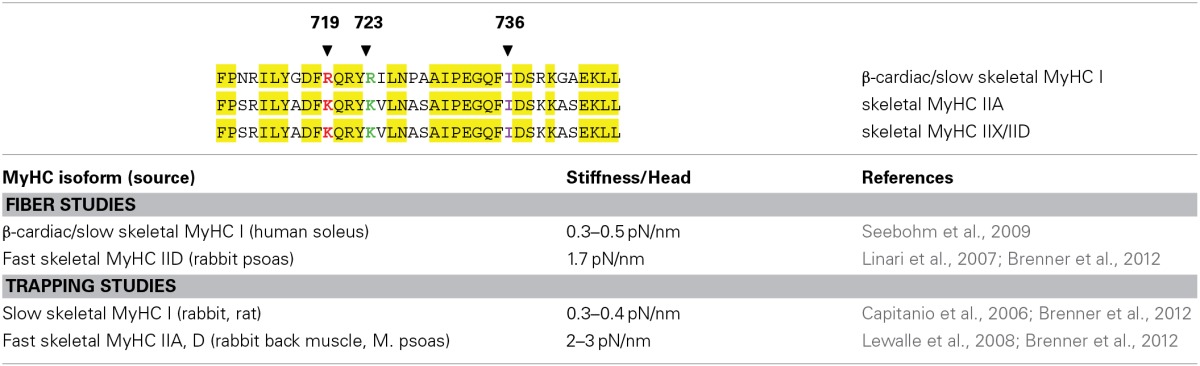
**Converter sequences and stiffness per myosin head for β-cardiac/slow skeletal and fast skeletal myosin heavy chain isoforms**.

Yellow, fully conserved residues, unmarked, residues where β-cardiac/slow skeletal myosin heavy chain sequence differs from the fast isoforms. Note that mutations R719W and R723G are located at positions where fast and slow isoforms differ. Mutation I736T is located at a position where fast and slow isoforms are identical. Stiffness per myosin head domain of fast and slow MyHC derived from stiffness measurements in muscle fibers and by optical trapping on myosin head domains (myosin subfragment 1) isolated from muscle tissue.

## Possible effects on cross-bridge cycling kinetics

To test whether (additional) effects on cross-bridge cycling kinetics are common to all three converter mutations and may be the common trigger of phenotype development, we measured the rate constant of force redevelopment (k_redev_) and fiber ATPase (Seebohm et al., 2009).

**Figure 4:**
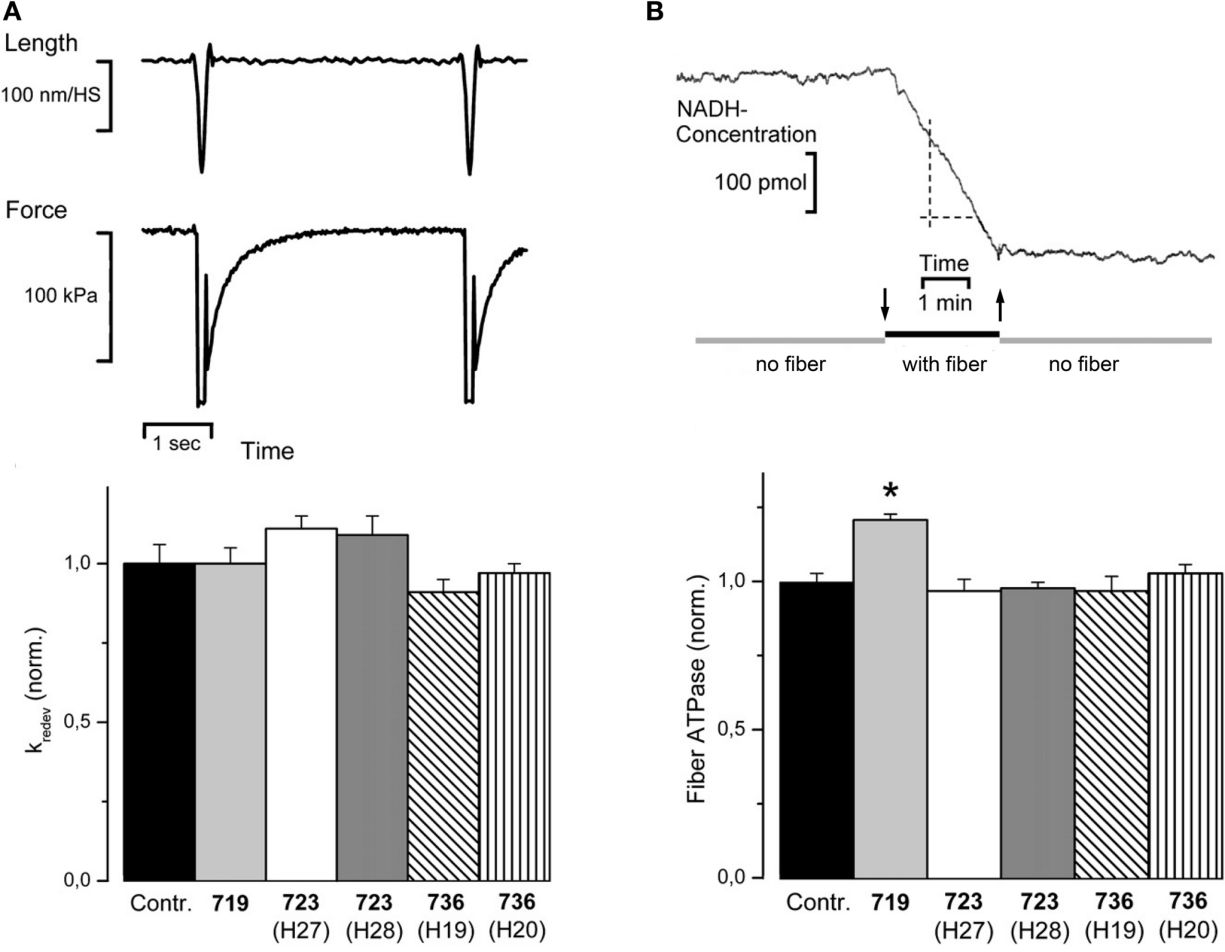
**(A)** Rate constant of force redevelopment (k_redev_). Top panel, original traces, fiber length, and isometric force vs. time; control fiber, *T* = 20°C. Release/restretch protocol to initiate force redevelopment (Brenner and Eisenberg, 1986). k_redev_ is the rate constant for the time course of force redevelopment to the isometric steady state, assuming a single exponential function. Isometric force is difference between force in isometric steady state and force level during the period of unloaded shortening where force has dropped to zero. Bottom panel, k_redev_ observed for mutations R719W, R723G, and I736T, normalized to k_redev_ of controls; *n* = 7–45 fibers. Mean values ± *SE*. No statistically significant effects could be observed. **(B)** Top panel, fiber ATPase, pen recorder trace of change in NADH-concentration while a single *M. soleus* fiber was incubated in the ATPase chamber. At downwards pointing arrow fiber was moved from trough with preactivating solution into ATPase chamber, at upwards pointing arrow fiber was moved back to preactivating solution. Maximum calcium activation, *T* = 20°C. ATPase is determined by the NADH-coupled enzyme assay in which rephosphorylation of ADP by phosphoenol pyruvate is coupled to reduction of pyruvate to lactate by NADH. Change in NADH concentration is followed by absorbance at 360 nm, calibrated with NADH test solutions. Bottom panel, effects of mutations R719W, R723G, and I736T, on fiber ATPase during isometric contraction (*n* = 6–21 fibers). *T* = 20°C. Mean values ± *SE* normalized to ATPase of control fibers. ATPase activity is significantly affected only by mutation R719W, indicated by ^*^p < 0.001 (reprinted from Seebohm et al., 2009 with permission from Elsevier).

Figure 4A shows original records of measurements of the rate constant of force redevelopment (top panel) together with the data obtained for the three converter domain mutations (bottom panel). No statistically significant effects were detectable for all three mutations. Figure 4B shows an original record of fiber ATPase measurements (top panel). These measurements revealed a statistically significant increase in ATPase for mutation R719W by about 20%. These ATPase measurements together with the unchanged rate constant of force redevelopment suggested some changes in cross-bridge cycling kinetics for the R719W mutation. A quantitative estimate, however, showed that the changes in cross-bridge cycling kinetics alone could at most account for about 1/3 of the increased active force by the resulting higher occupancy of force generating cross-bridge states during isometric steady state contraction and required the increased head stiffness to account for the overall increase in active force seen with mutation R719W. The increased stiffness in rigor, however, could not at all be accounted for by these changes in cross-bridge turnover kinetics (Seebohm et al., 2009).

**Figure 5:**
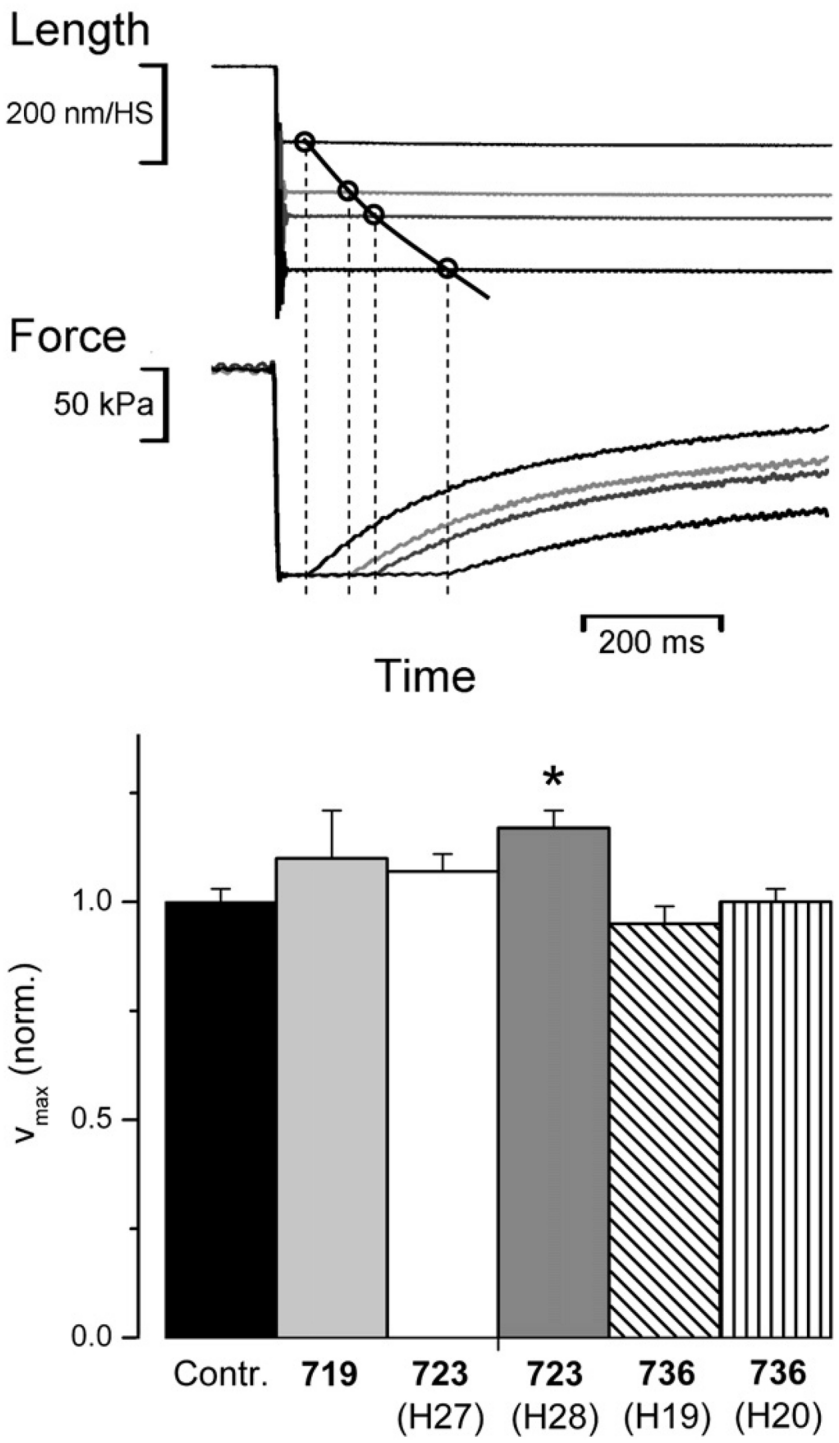
**Effect of converter mutations on maximum unloaded shortening velocity (v_max_)**. **Top panel**: sample traces of slack test experiment, length vs. time and force vs. time (cf. Edman, 1979). This illustrates the reconstruction of 4 points of unloaded shortening vs. time from the time required for unloaded shortening to take up the imposed slack, i.e., until force starts to redevelop; v_max_ defined as initial slope of the shortening curve is determined by curve fitting according to Brenner (1986). Control fiber at 20°C. **Bottom panel**, v_max_ for mutations R719W, R723G, and I736T normalized to control fibers; *n* = 6–11 fibers. *T* = 20°C; mean values ± *SE*. ^*^indicates statistically significant difference to controls (*p* ≤ 0.05). Reprinted from Seebohm et al. (2009) with permission from Elsevier.

To identify possible effects of the three converter mutations on cross-bridge kinetics under isotonic conditions we determined unloaded shortening velocity and force velocity relations on fibers from *M. soleus* samples of affected patients. Unloaded shortening velocity was determined by the slack-test experiment (Figure 5, top panel; cf. Edman, 1979). As we had previously shown, the time to shorten a preset distance (imposed fiber slack) vs. slack amplitude is not a linear relation as expected for a constant shortening velocity. Instead shortening velocity slows down with distance of shortening (Brenner, 1986). When the natural logarithm of velocity, however, was plotted vs. sarcomere length a linear relation was observed from which the shortening velocity at the very beginning of unloaded shortening could be determined (cf. Brenner, 1986). Data of unloaded shortening velocity determined from fibers of affected patients are shown in Figure 5 (bottom panel). Only fibers from one patient showed an increase in unloaded shortening velocity just above the significance cut-off of *p* < 0.05. All other data were not different from controls.

**Figure 6:**
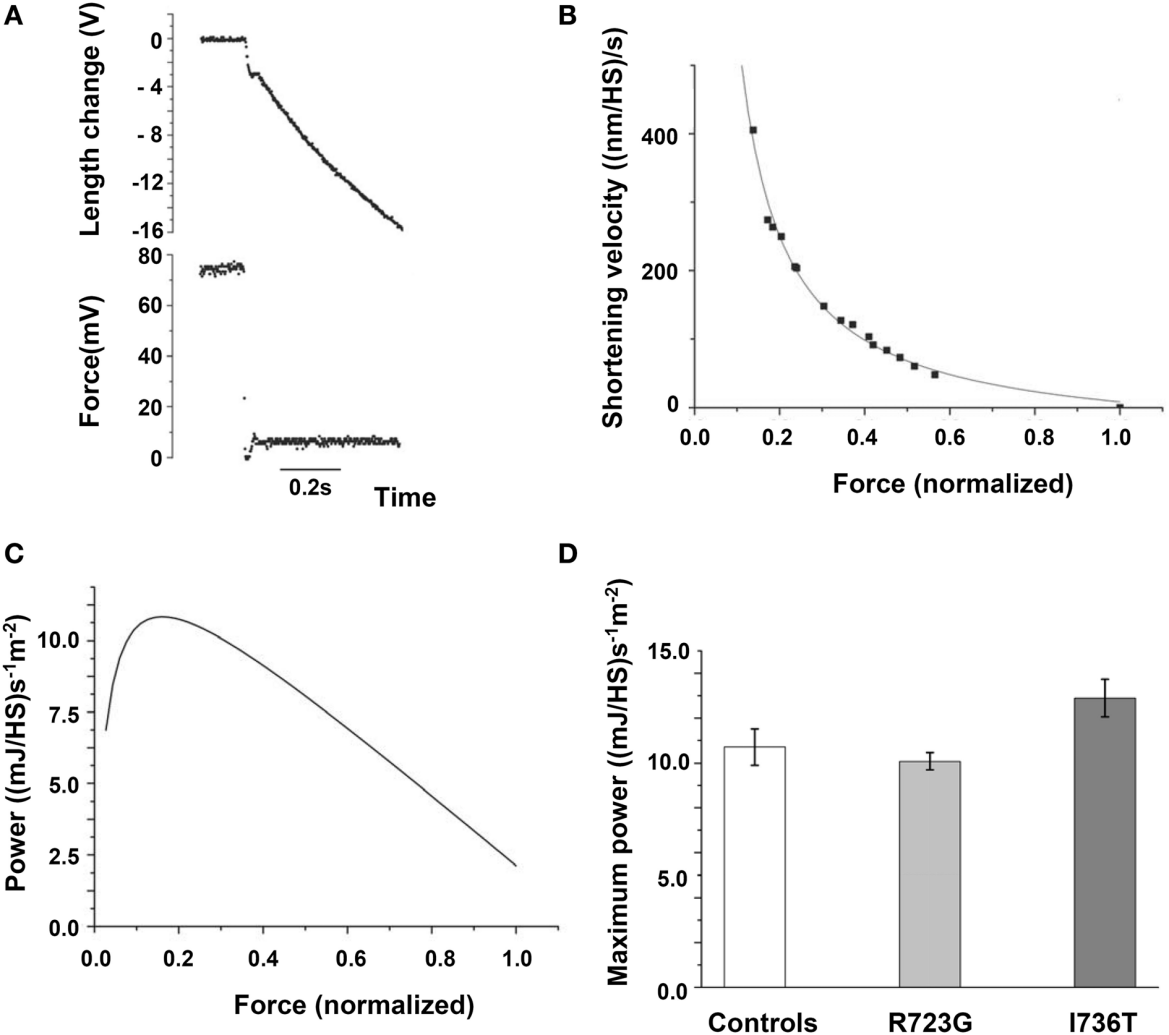
**Force-velocity relation and maximum power output**. **(A)** Original length and force traces of a quick release experiment in which the fiber was switched from isometric steady state to isotonic shortening. Load during isotonic shortening about 10% of isometric force. Note the curved time course of fiber length similar to the time course of unloaded shortening by the slack test experiment (Figure 5, top panel). Initial shortening velocity determined according to Brenner (1986). **(B)** Force-velocity relation constructed by plotting initial shortening velocity vs. applied load, i.e., force exerted by fiber during shortening. Solid line is fit of Hill equation (Hill, 1938) to data points. **(C)** Power at different loads, solid line derived from Hill equation fitted to data points, i.e., solid line in **(B)**. **(D)** Effect of mutations R723G and I736T on maximum power; mean ± *SE*, *n* = 12 − 14 fibers. Differences not statistically significant (*p* > 0.05). *T* = 20°C.

To test whether our FHC-mutations also significantly reduce maximum power under loaded shortening, as previously found for other FHC-mutations (Lankford et al., 1995), we determined isotonic shortening velocity under different loads for controls and mutations R723G and I736T. For this test, quick releases from isometric steady state contraction to shortening under load conditions were applied (Figure 6A). Initial shortening velocity at the different loads was determined by curve fitting according to Brenner (1986) just as done for unloaded shortening velocity. The velocity data were plotted vs. applied load (Figure 6B) and the Hill equation was fitted to the data points (Figure 6B solid line) to obtain the force-velocity relations. From the Hill equation we determined the power at different loads (Figure 6C) to determine whether the converter mutations affect the maximum power under isotonic conditions. As shown in Figure 6D, converter mutations R723G and I736T had no significant effect on maximum power under isotonic conditions. For mutation R719W force velocity relations could not be determined because of insufficient size of the biopsy to perform all different experiments. Nevertheless, neither unloaded shortening velocity nor the maximum power output could serve as parameters in which all three converter mutations consistently differ from controls.

## Force generation at different degrees of Ca^++^-activation; Ca^++^-sensitivity

**Figure 7:**
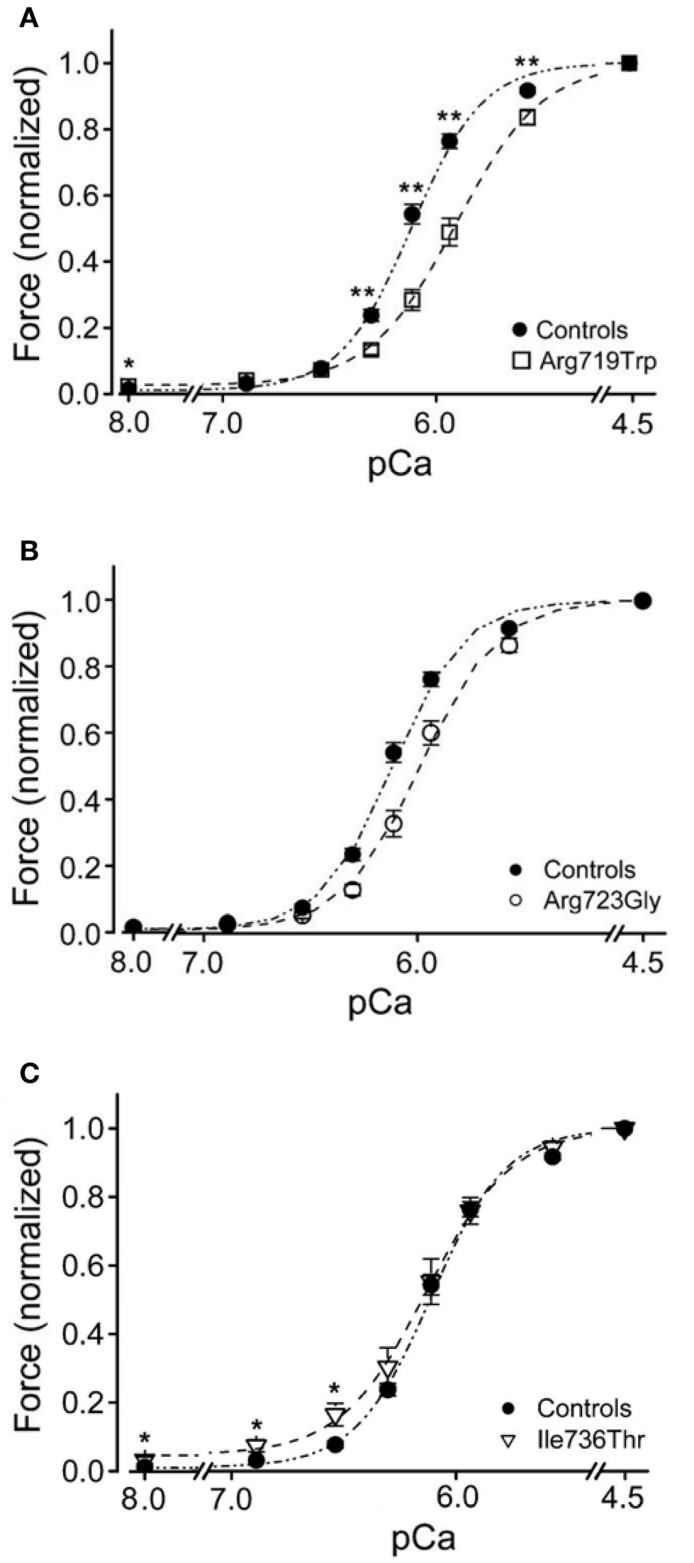
**Force-pCa relations of converter mutations vs. controls**. *N* = 19 control fibers. pCa_50_ of controls, 6.12 ± 0.02. **(A)** Mutation R719W; *n* = 16 fibers; ^**^ mark data at which difference in active forces are highly significant (*p* < 0.001); pCa_50_ of fibers with mutation R719W, 5.90 ± 0.03, significantly different from control (*p* < 0.001) **(B)** Mutation R723G, *n* = 13 fibers; pCa_50_ 5.98 ± 0.03, significantly different from control (*p* < 0.001). **(C)** Mutation I736T; *n* = 10 fibers; ^*^ mark data for which difference in force generation is significant (*p* < 0.01); pCa_50_ of fibers with mutation I736T, 6.15 ± 0.04, not significantly different from control (*p* > 0.05). Note, however, the incomplete relaxation even at pCa 8. All data recorded after treatment of fibers with PP1α; modified from Kirschner et al. (2005). With permission of The American Physiological Society.

It was previously reported that FHC-related mutations cause an increased Ca^++^-sensitivity of the contractile apparatus (Robinson et al., 2002; Ashrafian et al., 2011; Marston, 2011). We therefore measured active force-generation at different degrees of Ca^++^-activation and constructed the force-pCa relations. To exclude effects of different phosphorylation levels of the regulatory light chain of myosin (LC2S or MLC-2 fast; Sweeney et al., 1993) all fibers were dephosphorylated with protein phosphatase 1α (Kirschner et al., 2005). The recorded force-pCa relations are shown in Figure 7. Somewhat surprisingly we found for converter mutations R719W and R723G a reduced Ca^++^-sensitivity. For mutation I736T Ca^++^-sensitivity was essentially unchanged but we found residual active force generation even under relaxing conditions (Kirschner et al., 2005).

### Implications of the functional effects observed with the three converter mutations

Converter mutations R719W and R723G, both located in the core of the converter at positions where slow and fast myosins differ in amino acid sequence, increase head stiffness. This implies that the converter is a main determinant of head stiffness and thus of the contribution of a myosin head to force generation. Mutation I736T, located at the surface of the converter has no such effect. The other surprising effect is that both R719W and R723G, opposite to the expectation for FHC-related mutations (Marston, 2011) reduce Ca^++^-sensitivity while mutation I736T has no effect on Ca^++^-sensitivity but causes an incomplete relaxation. In addition, mutation R719W showed some changes in cross-bridge cycling kinetics. Altogether, up to this point, the three converter mutations did not show a common direct functional effect. Thus, whilst increased head stiffness is a direct functional effect of the FHC-mutations R723G and R719W, the FHC-phenotype cannot simply be the result of increased head stiffness. Interestingly, higher head stiffness is in fact normal for fast skeletal muscle myosin when compared with the slow skeletal isoform (Table 1). So how does increased head stiffness in the β-myosin heavy chain (β-MyHC) cause disease in myocardium?

### How may mutations in the myosin head domain trigger development of an FHC-phenotype?

**Figure 8:**
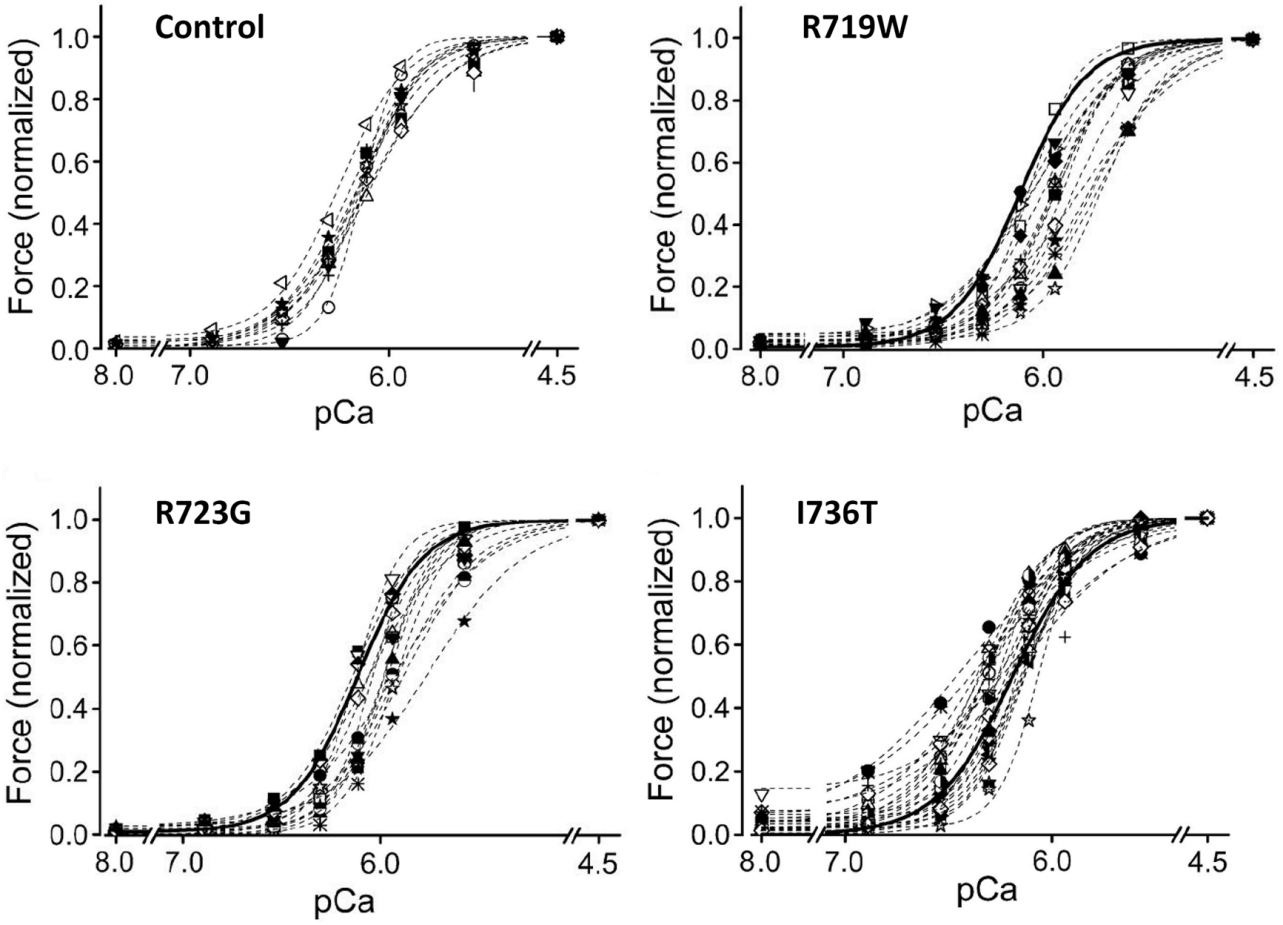
**Force-pCa relations of individual *M. soleus* fibers**. Controls, 10 fibers of a control individual; R719W, 16 fibers (cf. Figure 7A); R723G, 13 fibers (cf. Figure 7B); I736T, 25 individual fibers. Heavy solid lines in plots of fibers with mutations represent the average force-pCa relation of the control fibers. Note that for all three mutations the individual force-pCa relations represent a continuum from relations like control fibers to force-pCa relations substantially shifted beyond the average position of the corresponding mutation shown in Figure 7, i.e., to the right for mutations R719W and R723G, and upwards to incomplete relaxation for mutation I736T. Modified from Kirschner et al. (2005). With permission of The American Physiological Society.

A possible insight into the puzzle of how FHC-related mutations in the myosin head domain with different direct functional effects may cause development of an FHC-phenotype was revealed when we compared force-Ca^++^-relationships of individual *M. soleus* fibers of FHC patients. To our surprise, in some fibers the force-pCa relations were just like those in unaffected controls while other fibers showed substantially different force-pCa relations (cf. Figure 8, Kirschner et al., 2005).

For mutation R719W force-pCa relations of some fibers were shifted substantially further to the right than expected from the “average” fiber (Figure 8 vs. Figure 7A). Similarly, some fibers with mutation R723G were also shifted much further to lower Ca^++^-sensitivity than the average fiber (Figure 8 vs. Figure 7B). Some fibers with mutation I736T showed substantially increased forces at partial activation and had still substantial active forces even at relaxing Ca^++^-concentrations, i.e., showed incomplete relaxation while other fibers of the same tissue sample had force-pCa relations indistinguishable from control fibers. The common feature of all three FHC-mutations, however, is the spectrum of force-pCa relations ranging from normal (control) to substantially shifted to “abnormal” Ca^++^-sensitivity or enhanced partial activation with incomplete relaxation.

The relevance of such spectrum of different Ca^++^-sensitivities for fibers of the same tissue sample of an individual patient becomes most obvious when considering force generation at partial activation. At Ca^++^-concentrations resulting in control fibers in about 60% of full activation, e.g., pCa 6.1, the observed active force generated by individual control fibers varies between about 50% and 70% of full activation. Thus, in control fibers the highest observed force levels observed at pCa 6.1 (70% of full activation; Figure 8) are about 1.5 times higher than the lowest (50% of full activation). At the same pCa value, about 6.1, for mutations R719W and R723G the highest forces of individual fibers in Figure 8 are at least 4- to 5-fold larger than the lowest. For mutation I736T the difference between fibers with highest forces vs. fibers with lowest forces at pCa 6.1 are again in this range (Figure 8). In addition, for mutation I736T some fibers still generated substantial forces at relaxing Ca^++^-concentrations where control fibers were fully relaxed (pCa 8.0).

Thus, the common feature for all three FHC-related mutations in the converter is the much larger spectrum of forces generated by individual fibers of one and the same patient at partial activation levels. In skeletal muscle such different force generation among individual fibers at partial activation means that individual fibers contribute differently to the force generated by a whole muscle. Yet, “stronger” or “weaker” fibers are not expected to interfere with each others function. This is because skeletal muscle fibers do not form cellular networks but contribute to total force of a muscle independently.

### What may be the reason for the variance from normal to much altered Ca^++^-sensitivity among individual fibers of the same FHC-patient?

**Figure 9:**
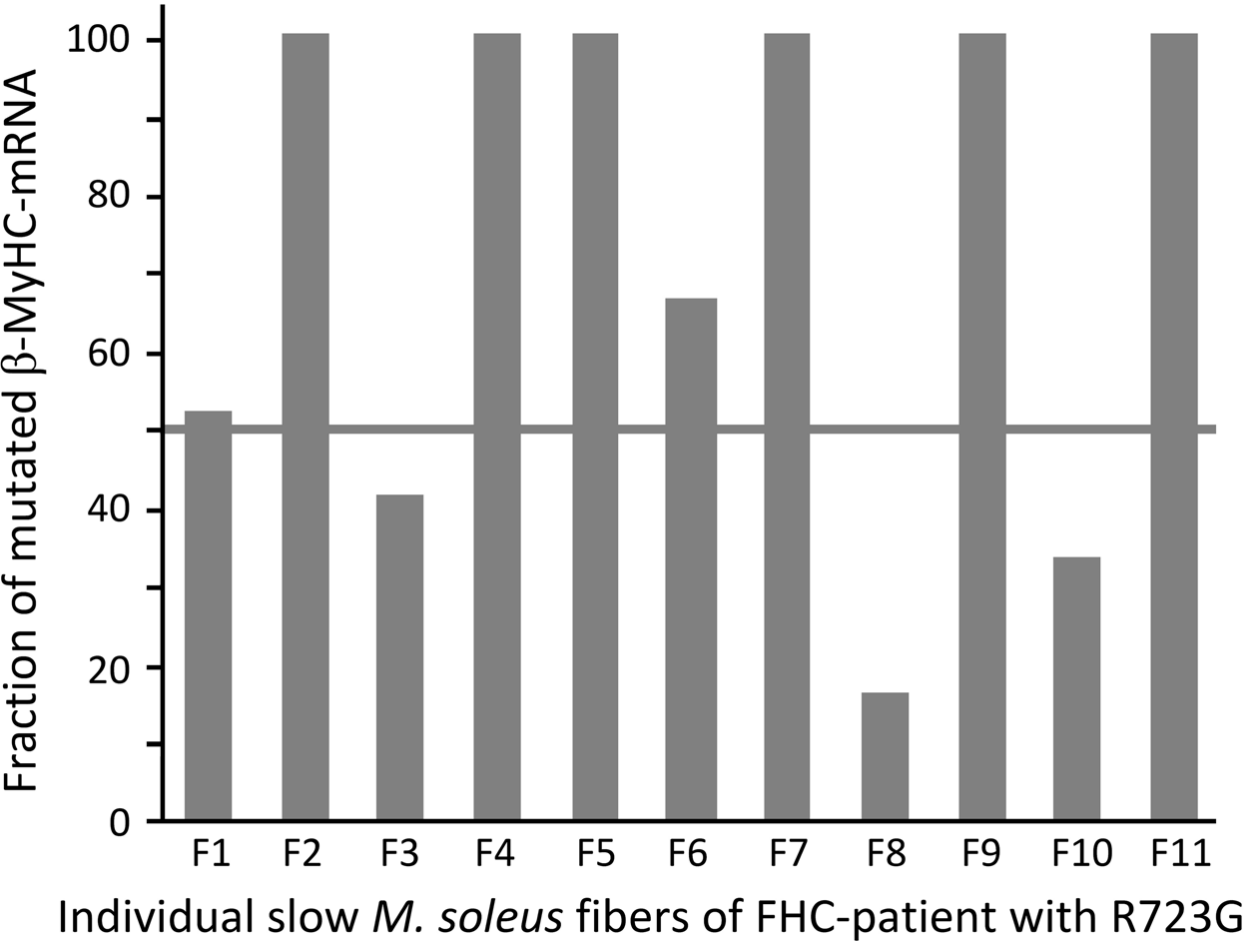
**Fraction of mutated β-cardiac/slow skeletal MyHC-mRNA in 11 individual fibers of *M. soleus* of a patient with mutation R723G**. A fiber bundle of *M. soleus*, flash-frozen without prior skinning, was freeze dried for isolation of individual fibers (Stienen et al., 1983) without cross-contamination of mRNA from fiber to fiber. For quantification of the fraction of mutated β-cardiac/slow skeletal MyHC-mRNA, isolated segments (2–3 mm) of individual fibers were processed as previously described for *M. soleus* fiber bundles (Tripathi et al., 2011). Note the large variation in the fraction of mutated β-cardiac/slow skeletal MyHC-mRNA among individual fibers.

We had previously observed that in soleus muscle samples of FHC-patients mutant and wildtype β-cardiac/slow skeletal MyHC were not expressed equally, different to what one may have expected for heterozygous patients with an autosomal dominant disease. Instead each mutation has a characteristic fraction of mutant β-cardiac/slow skeletal MyHC both at the mRNA and protein level (Figure 3, Tripathi et al., 2011). We therefore wondered whether the fraction of β-cardiac/slow skeletal MyHC may also vary from fiber to fiber. Because of the very small amount of material obtainable from individual soleus fiber segments we could only determine the fraction of mutant β-cardiac/slow skeletal MyHC at the mRNA level. Individual *M. soleus* fibers of a patient with mutation R723G showed quite different fractions of mutant mRNA, ranging from about 10% to essentially 100% (Figure 9). The high number of fibers with almost 100% mutant mRNA is consistent with the observation that in soleus samples (tissue level) the average fraction of mutated mRNA is ≥2/3 of total β-cardiac/slow skeletal MyHC-mRNA (cf. Figure 3).

In tissue samples of FHC-patients we always found a close correlation between fraction of mutant mRNA and fraction of mutant protein, regardless of the FHC-mutation we studied (Figure 3, Tripathi et al., 2011). Thus, the observed large variation in the fraction of mutant mRNA among individual fibers of soleus muscle of affected patients very likely correlates with a similar variation in the fraction of mutant protein. The large variation in mutant mRNA and thus in mutant protein among individual soleus fibers of affected patients could well account for the large functional variation among individual fibers, as judged by the force-pCa relations and the large variation in active force at partial activation (cf. Figure 8, Kirschner et al., 2005).

Assuming that the fibers with the largest shift in force-pCa relations have the highest abundance of mutant β-cardiac/slow skeletal MyHC, e.g., 100% as the maximum, forces generated by these fibers would at most be 2-fold larger than force of control fibers. This is because force generation per head with mutations R719W or R723G was estimated to be about 2-fold higher than force generation by wildtype myosin heads; see above (Kirschner et al., 2005). As a consequence, the forces at partial activation (pCa ≥ 6.3), as low as 1/5 of control fibers in the normalized plots (Figure 8), would still be less than half of control in absolute terms. At pCa around 5.8–5.9 fibers with mutant myosin are expected to generate about the same amount of absolute force as controls, at higher activation levels fibers with mutations R719W or R723G would generate higher forces than controls, reaching up to 2-fold higher forces at maximum activation for fibers with 100% mutant myosin. Most importantly, however, even if absolute forces of individual fibers are considered, the large variation in Ca^++^-sensitivity among individual fibers remains unaffected.

### In myocardium direct functional effects can be masked or even reversed by secondary effects

Characterization of functional effects of FHC-related mutations in myocardium for comparison with the effects seen in *M. soleus* fibers became possible by samples from explanted hearts of patients with the myosin missense mutations, R723G, one of the mutations that we had studied on soleus muscle samples.

**Figure 10:**
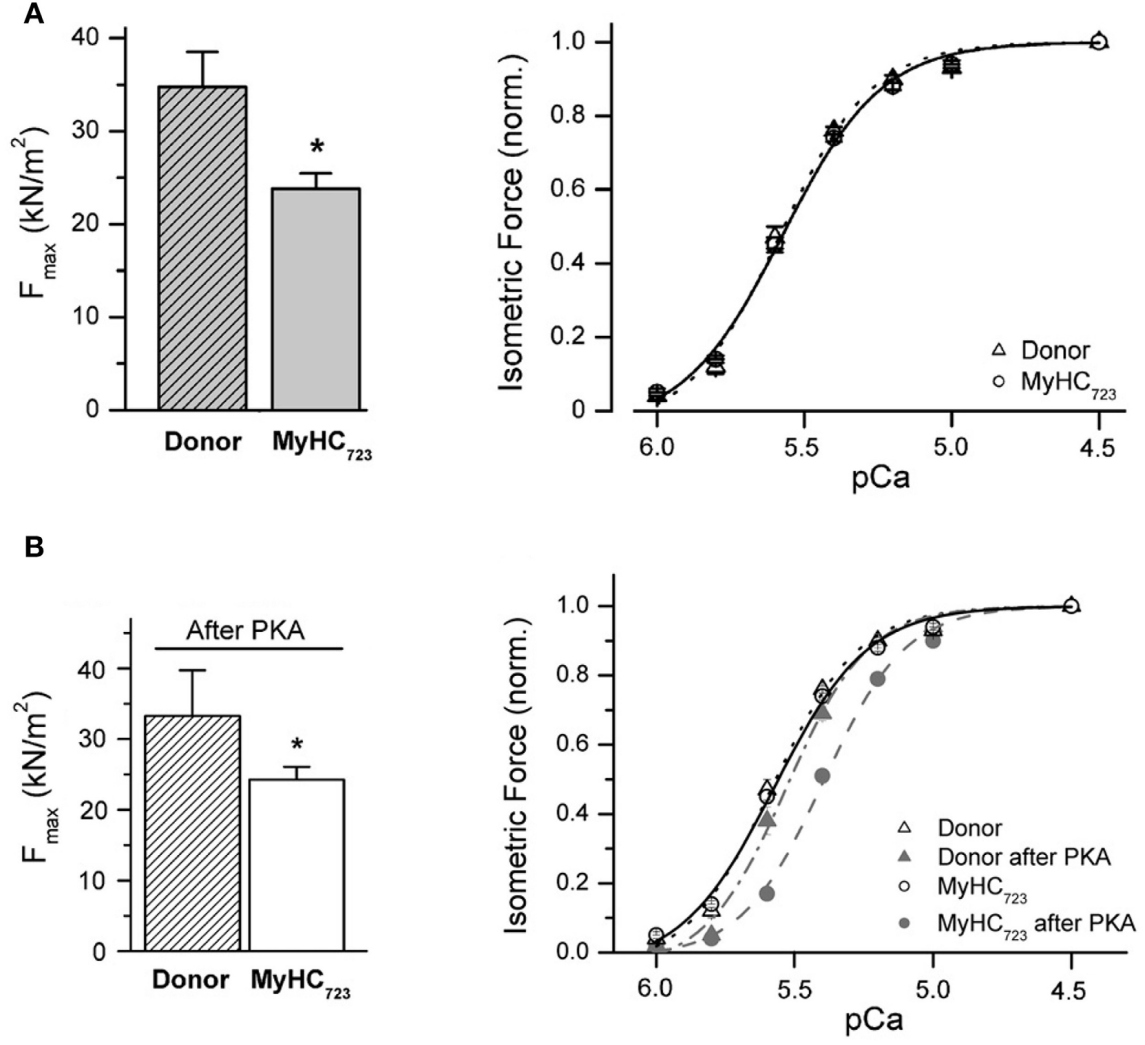
**Effect of mutation R723G in myocardium**. **(A)** Isolated cardiomyocytes, native state of phosphorylation. Left panel, maximum active force per cross sectional area at saturating calcium-concentration (F_max_). F_max_ of MyHC723-cardiomyocytes (light gray bar) is 35% lower than F_max_ of donor cells (hatched bar; ^*^p < 0.05). Right panel, force-pCa relation normalized to maximum force at saturating Ca^++^-ion concentration (pCa 4.5). Solid and dotted lines, fits of a modified Hill equation yielding pCa_50_. Note that opposite to *M. soleus* fibers, cardiomyocytes isolated from a tissue sample of an explanted heart show a decrease in maximum active force with no shift in the force-pCa relation. **(B)** Isolated cardiomyocytes after incubation with protein kinase A (PKA) to match phosphorylation of contractile proteins, particularly of cTnI. Left panel, F_max_ after treatment with PKA ^*^p < 0.05. Note that PKA-treatment does not change the difference in F_max_ between R723G-cardiomyocytes and controls. Right panel, force-pCa relations of MyHC723-cardiomyocytes (gray filled circles, dashed line) vs. donor cardiomyocytes (gray filled triangles, dashed-dotted line). Note the shift of the force-pCa relation of MyHC723-cardiomyocytes vs. donor after PKA treatment. For comparison force-pCa relations obtained before PKA treatment are also plotted in the same graph (open circles and solid line, MyHC723-cardiomyocytes; open triangles and dotted line, controls). Lines are fits of modified Hill equation to data points. Note that after PKA-treatment to match phosphorylation of e.g., cTnI, a similar reduced Ca^++^-sensitivity becomes detectable for MyHC723-cardiomyocytes just like that seen in the soleus fibers. Modified from Kraft et al. (2013). With permission of the Journal of Molecular and Cellular Cardiology.

Cardiomyocytes were mechanically isolated from the flash-frozen tissue samples (van der Velden et al., 2003; Kraft et al., 2013). After chemical permeabilization the isolated cardiomyocytes were attached to a force transducer and motor such that active and passive forces and kinetics of cross-bridge cycling (k_redev_) could be measured at different concentrations of free Ca^++^-ions. To our surprise, force-pCa relations of cardiomyocytes isolated from the flash-frozen samples of explanted myocardium of two patients with mutation R723G showed (i) no difference in Ca^++^-sensitivity and (ii) reduced maximum active force compared to myocytes from donor hearts (Figure 10A). This was in total contrast to the reduced Ca^++^-sensitivity and increased maximum force seen in soleus samples with the same mutation.

Analysis of phosphorylation of contractile proteins revealed substantially lower phosphorylation of troponin I in these myocardial samples with mutations compared to the controls. When the phosphorylation pattern was matched with controls by treatment with protein kinase A (PKA) or by a combination of dephosphorylation by protein phosphatase-1α (PP-1α) followed by phosphorylation with PKA, the mutant samples showed a similarly reduced Ca^++^-sensitivity as the soleus fibers of patients with the R723G-mutation (Figure 10A vs. Figure 10B).

Maximum force, however, was still substantially reduced after matching phosphorylation of patient samples with controls (Figure 10B, left panel). An explanation for the reduced force generation in myocardium in contrast to the increased force generation in soleus fibers with the same mutation was revealed by electron microscopy of tissue samples of affected patients. In myocardium of affected patients prominent intracellular myofibrillar disarray and reduced packing of myofibrils within cardiomyocytes was observed with non-myofibrillar and non-mitochondrial material taking up substantial intracellular volume (Kraft et al., 2013). Quantitatively, myofibrillar density of cardiomyocytes of affected patients with mutation R723G was reduced by about 26%. Without such reduced myofibrillar density within cardiomyocytes, force generation per cross-sectional area had been essentially the same as in control samples (Kraft et al., 2013). The additional myofibrillar disarray with myofibrils deviating from the longitudinal axis of a cardiomyocyte will add to reduced force generation in axial direction (Friedrich et al., 2010) such that increased force generation is not observed even when accounting for reduced myofibrillar density.

Altogether, the opportunity to compare effects of the R723G missense mutation in both soleus fibers and myocardium of affected patients revealed that at least at stages of disease development when myectomies were taken or when transplantation was necessary, direct functional effects of FHC-related mutations can be masked or even reversed by effects most likely subsequent to the direct functional effects. This suggests that e.g., FHC-typical cardiomyocyte and myofibrillar disarray are not direct results of FHC-related mutations but develop subsequently as a result of functional alterations. Functional effects of different mutations apparently converge to a common path of changes resulting in the FHC-phenotype. Cardiomyocyte and myofibrillar disarray may represent the start of this common path leading to e.g., interstitial fibrosis and hypertrophy.

So what may be a feature common to different FHC-related mutations that could trigger cardiomyocyte and myofibrillar disarray as the start on a common path to an FHC-phenotype?

## Hypothesis for development of an FHC-phenotype in myocardium

In our previous work on FHC-related missense mutations in the β-cardiac/slow skeletal MyHC we made three key observations. (i) A large variation in calcium sensitivity among individual *M. soleus* fibers of FHC-patients with mutations R723G, R719W, and I736T. This variation ranged from essentially normal calcium sensitivity to highly different, e.g., reduced calcium sensitivity for mutations R719w and R723G. (ii) The ratio of mutated vs. wildtype β-cardiac/slow skeletal MyHC is not 1:1 but characteristic for each mutation. In addition this ratio is very similar at both the mRNA and protein level. (iii) Among individual fibers of *M. soleus* samples with the R723G mutation, the ratio of mutant vs. wildtype mRNA varies from almost pure mutant to almost pure wildtype. A substantial variation in the ratio of mutant vs. wildtype protein from fiber to fiber was previously also found for the R403Q mutation in the β-cardiac/slow skeletal MyHC (Malinchik et al., 1997).

**Figure 11:**
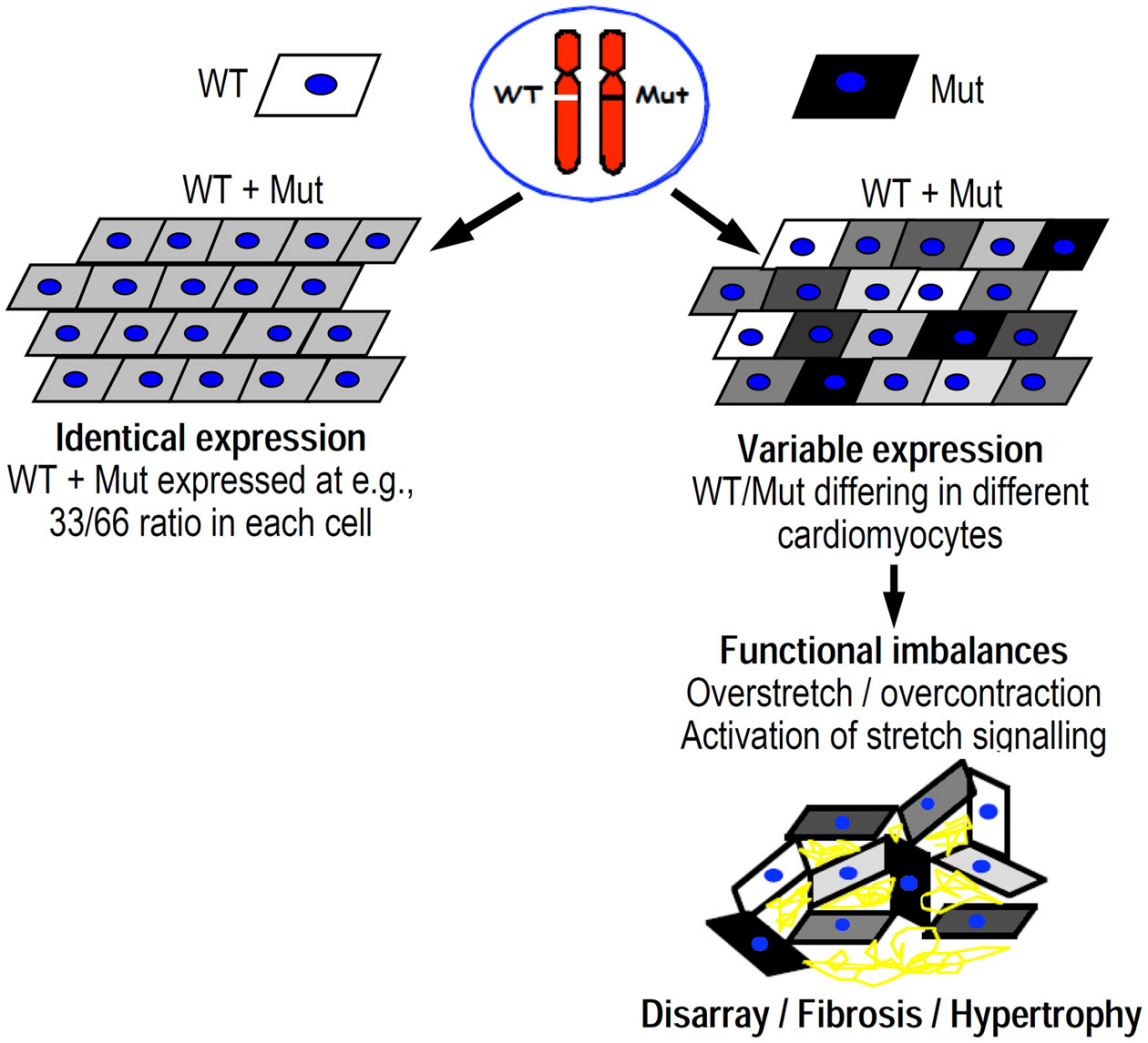
**Scheme illustrating the difference between equal abundance of mutant protein in all cardiomyocytes (top left) vs. variation in abundance among individual cardiomyocytes (top right)**. Gray level illustrates abundance of mutant protein. Unequal abundance of mutant protein in individual cardiomyocytes results in functional imbalance that distorts the cellular network of the myocardium (bottom right). Overstretch vs. overcontraction results in highly variable activation of e.g., stress/strain signaling with development of disarray, hypertrophy, and interstitial fibrosis in the long run.

**Figure 12:**
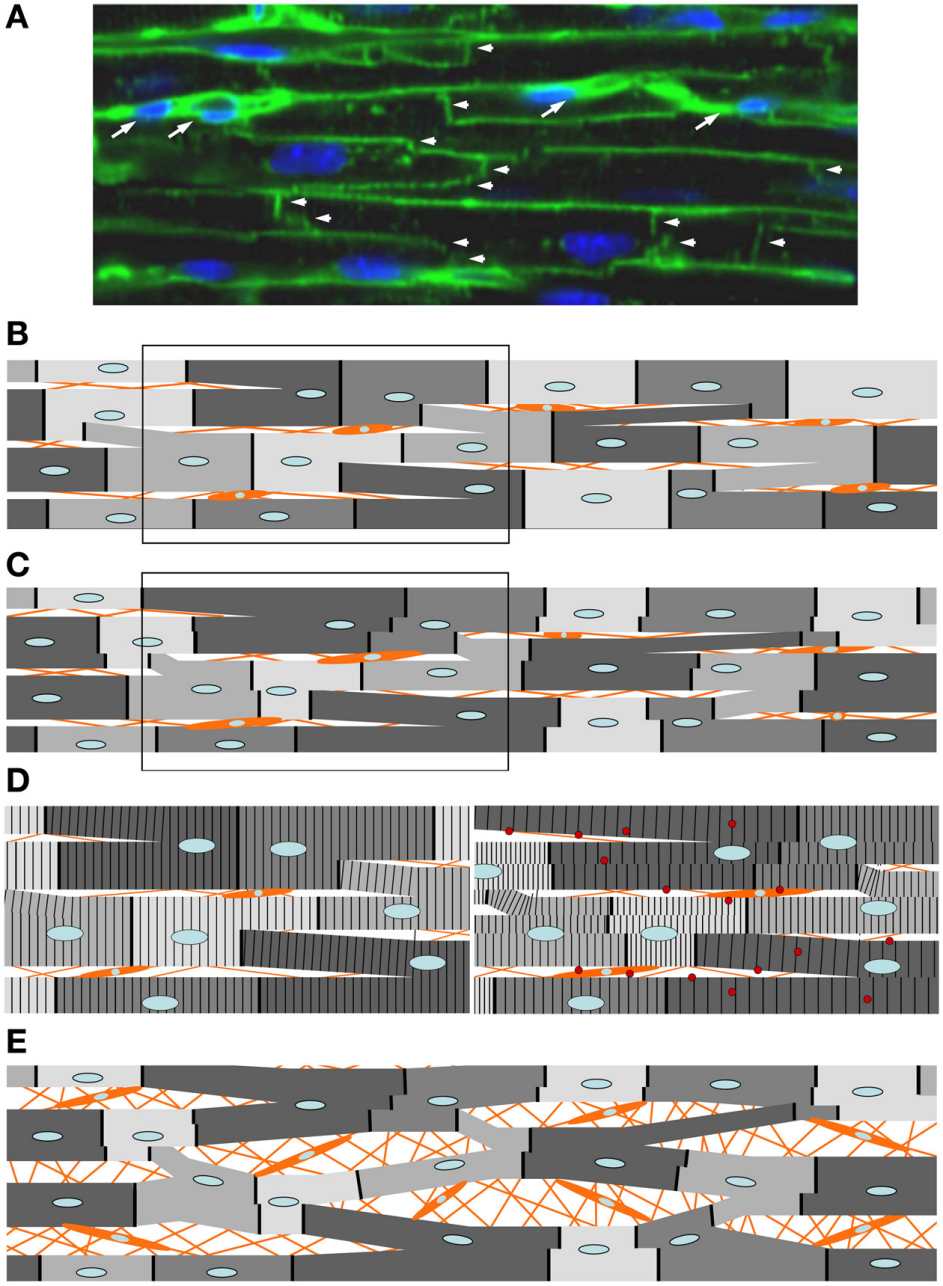
**Expected response of the cellular network of myocardium to imbalances in force generation among individual cardiomyocytes, e.g., by mutations R719W or R723G**. Orange lines and orange cells represent extracellular connective tissue and non-myocyte cells within the myocardium. **(A)** Confocal image of a longitudinal section through myocardium (adult mouse). Intercalated discs (vertical lines and “staircases” labeled with arrow heads) labeled with a mouse monoclonal antibody to pan Cadherin and an Alexa 488-linked goat-anti-mouse antibody. Horizontal lines are plasma membranes visualized by staining glycoproteins containing β-N-acetyl-D-glucosamine with FITC-conjugated wheat germ agglutinin (for details see Schipke et al., in press). TO-PRO®-3 Iodide used for staining nuclei (blue). By this approach, individual cardiomyocytes are delineated. Note that individual cardiomyocytes are not arranged in independent columns. Instead, cardiomyocytes make contact via intercalated discs with cardiomyocytes of adjacent columns resulting in a cellular network. Presumed non-myocyte cells are labeled with white arrows. **(B)** Schematic representation of the cellular arrangement in myocardium with branched cardiomyocytes while relaxed. The darker the gray-level the higher the abundance of the mutant myosin. The mutation is assumed to result in substantially lower forces at partial activation levels despite higher force at maximum activation, as was seen for mutations R719W and R723G (cf. Figure 8) **(C)** Same cellular network during contraction (partial activation like in a twitch). For cardiomyocytes with mutations R723G or R719W higher abundance of the mutant myosin (darker gray levels) results in lower forces at partial activation. Thus, during a twitch these cardiomyocytes become overstretched by those with low abundance of the mutant protein (lighter gray levels). Due to the branching, different parts even of individual cells will experience different forces by the different connections with neighboring cells and will therefore shorten or be stretched to different extent (see steps in intercalated discs, represented by dark solid vertical lines separating adjacent cells). Due to relative movement of cells in parallel strands, branches between the strands will be distorted as will be the non-myocyte cells in between the strands of cardiomyocytes. **(D)** Left panel, boxed part of **(B)** magnified to illustrate striation pattern; right panel, boxed part of **(C)**. Note the developing differences in sarcomere lengths of myofibrils during contraction even within an individual cell. This is due to the branching of the cells and the different forces acting on the branches due to the unequal force generating capability of adjacent cells by the different abundance of mutant protein (functional imbalance). Thus, functional imbalance among individual cardiomyocytes together with the cellular network of the cardiomyocytes are expected to trigger cellular and myofibrillar disarray whenever, by variation in expression, a mutation causes unequal force generation among individual cardiomyocytes during a twitch. This state would represent the profibrotic state of the myocardium (Ho et al., 2010) in which stretch triggers increased expression of e.g., Tgf-β (red spheres) by cardiomyocytes and non-myocyte cells activating development of interstitial fibrosis. Such distortion will take place during force generation (pressure development) and will be enhanced during shortening under load (ejection period). This will occur even if maximum shortening velocity is unaffected by the mutation because the relative load will be lower for “strong” cells (low abundance of mutant protein) vs. “weak” cells (high abundance of mutant protein). Note, that if the mutant protein were expressed equally in all cells, force generation at any time during a twitch would be affected equally and no or only minimal changes in the arrangement of cardiomyocytes would take place during a twitch, i.e., no or only much smaller changes from the arrangement in **(B)** would be seen. Panel **(E)** illustrates a later stage of phenotype development when e.g., stretch-sensitive signaling has triggered development of interstitial fibrosis with increased cellular disarray. The increase in cell size by signaling paths that trigger hypertrophy is not shown.

Based on these three observations, we proposed the following hypothesis for a common trigger of the FHC-phenotype development (Figure 11, Kirschner et al., 2005; Tripathi et al., 2011):

- Variation of the fraction of the mutated protein among individual cardiomyocytes (different gray levels of schematic cardiomyocytes in Figure 11), just as seen among individual *M. soleus* fibers, results in functional imbalances, e.g., unequal force generation particularly at low activation levels (cf. Figure 8) among the individual cardiomyocytes.
- Since cardiomyocytes, different from skeletal muscle fibers, are branched and form a cellular network (Figure 12A), such variance in force generation among individual cells will result in uneven contraction, i.e., over-contraction vs. overstretch of cardiomyocytes during each twitch. Such functional imbalance will not only occur during force generation (pressure development in the ventricles) but also during shortening under load (ejection period). This is because for cells generating higher forces the relative load during ejection period will be lower compared to cells generating lower forces, even if v_max_ is unaffected by a mutation. As a result, over-contraction of some cells while others are overstretched will distort the myocyte network at the cellular and myofibrillar level (Scheme in Figure 12B vs. Figure 12C, and left panel in Figure 12D vs. right panel).
- The structural disorganization, even if initially only a transient feature during each twitch, however, will result in progression of persisting structural disorganization over months and years because of the triggering of secondary changes. Structural distortion could, for example, trigger stretch sensitive signaling, e.g., Tgf-β signaling by cardiomyocytes and non-myocyte-cells (right panel in Figure 12D) that was shown to be critical for pathologic remodeling of the myocardium in FHC (Teekakirikul et al., 2010). Stretch induced increase in Tgf-β was found in cell cultures both for cardiomyocytes and non-myocyte-cells (Ruwhof et al., 2000; van Wamel et al., 2001, 2002). In our hypothesis, structural disorganization of the cellular network of the myocardium because of functional imbalance among individual cardiomyocytes initiates, via stretch-sensitive signaling paths, cardiac remodeling (Figure 12E) with interstitial fibrosis, cellular disarray and hypertrophy, i.e., hallmarks of the FHC-phenotype (Ho et al., 2010). Increased collagen synthesis in a profibrotic myocardial state (Ho et al., 2010), supports our concept.

Based on this concept, for prevention of FHC-phenotype development therapeutic interventions are required that affect myocardial function differentially, e.g., specifically the altered function of the mutant protein, otherwise the functional imbalance will not be reduced and cardiac remodeling will not be prevented. Alternatively, inhibition of subsequent, e.g., stretch-induced signaling that initiates interstitial fibrosis could be a therapeutic target to prevent fibrosis and cardiac remodeling. This target was recently addressed by Teekakirikul et al. (2010).

Since skeletal muscle fibers are only very rarely branched, a similar extensive disarray is not expected and not observed in *M. soleus* fibers. Central core disease, found in soleus fibers of some FHC-patients are localized changes inside the soleus fibers with absence of mitochondria, smaller myofibrils, some overcontracted sarcomeres and irregular Z-discs (Fananapazir et al., 1993). This, however, is different from the extensive disarray seen in myocardium and may suggest unequal abundance of mutated protein along skeletal muscle fibers. Overcontracted sarcomeres and irregular Z-discs may not be unexpected since skeletal muscle fibers have multiple nuclei. Thus, variation in the expression of mutant vs. wildtype mRNA and protein among different nuclei may exist within skeletal muscle fibers and thus cause functional imbalances along these fibers.

In our hypothesis, any functional imbalance among individual cardiomyocytes, resulting from e.g., variable abundance of a mutant protein, has the potential to induce an FHC-phenotype. This could include not only missense mutations that affect force output of cardiomyocytes (poison peptide principle), but also the expression and degradation of truncated proteins, resulting in variable amounts of normal protein (principle of haplo-insufficiency). In principle, non-sarcomeric proteins that affect contractile function could also trigger FHC-phenotype development if unequal effects are generated among individual cardiomyocytes, e.g., by variation in expression of the mutant protein. In homozygous patients, found only very rarely, functional imbalance is not expected and the disease is dominated by direct functional effects of the mutations. These are always present and lead to the functional imbalance in the heterozygous patients when mutant protein varies from cell to cell.

## Testing the hypothesis

Our hypothesis is based on the assumption that among individual cardiomyocytes a similar variation in the expression of mutant vs. wildtype mRNA exists as shown in Figure 9 for *M. soleus* fibers, and that this results in functional variation, i.e., imbalances in force generation and shortening among neighboring cardiomyocytes. Thus, quantification of mutant vs. wildtype mRNA in individual cardiomyocytes is a critical test for our hypothesis. First trials of such quantification of mutant vs. wildtype mRNA in individual cardiomyocytes indicate that indeed the expression of mutant mRNA varies among individual cardiomyocytes from near zero to almost pure mutant mRNA (Montag et al., 2014).

## In summary

Our functional studies on tissue samples of FHC-patients showed that missense mutations in the myosin head domain do not result in a direct functional effect common to all mutations like increased force generation, increased Ca^++^-sensitivity, or increased ATPase. One feature common to all mutations we have studied is a large variation in the force-pCa-relation among individual *M. soleus* fibers from normal to highly different. As a consequence, particularly at partial activation large differences in the generated active forces exist among individual fibers. Quantification of mutant mRNA suggests that this functional variation is due to variation in the fraction of mutant β-cardiac/slow skeletal MyHC present in individual fibers. If such functional imbalance among individual cells exists in a cellular network like the myocardium, the functional imbalance will result in distortions of cells within this network. We hypothesize that such structural distortions result in myocyte and myofibrillar disarray and activate stretch-induced signaling, e.g., Tgf-β-signaling, that initiates cardiac remodeling with interstitial fibrosis and hypertrophy, the structural hallmarks of FHC. On this basis, any mutation in a sarcomeric or non-sarcomeric protein that results in similar functional imbalance among individual cardiomyocytes has the potential to trigger development of an FHC-phenotype. Such a mechanism as the trigger of FHC-development would have fundamental implications for therapeutic strategies. For such a pathomechanism either mutation-selective interventions are needed to ameliorate functional imbalances among individual cardiomyocytes to prevent FHC-phenotype development. Alternatively, inhibition of stretch-induced signaling, e.g., Tgf-β-signaling could be another target to prevent cardiac remodeling in FHC.

### Conflict of interest statement

The authors declare that the research was conducted in the absence of any commercial or financial relationships that could be construed as a potential conflict of interest.
